# The Lifetime Dance Exposure Questionnaire for Professional Training: Survey Development and Reliability Study

**DOI:** 10.2196/75632

**Published:** 2026-03-27

**Authors:** Aston K McCullough, Kai Yi (Kaye) Han

**Affiliations:** 1 Laboratory for the Scientific Study of Dance, Institute for Cognitive and Brain Health, Northeastern University, 360 Huntington Ave, 129 ISEC, Boston, MA, 02115, United States, 1 6173738893; 2 Department of Physical Therapy, Movement and Rehabilitation Sciences, Northeastern University, Boston, MA, United States; 3 Department of Music, Northeastern University, Boston, MA, United States

**Keywords:** survey, test-retest reliability, physical activity, self-report, adult

## Abstract

**Background:**

Dance is a complex mode of physical activity (PA) behavior and an art form, and one’s participation in dance may occur across discrete contexts throughout the lifespan. To further advance the study of lifetime participation in dance behavior, reliable questionnaires are needed.

**Objective:**

This study aimed to develop and test the reliability of a self-report, online questionnaire for surveilling lifetime participation in professionally led dance classes among adults.

**Methods:**

Community-dwelling adults (N=373) completed the Lifetime Dance Exposure Questionnaire for Professional Training (LDEQ-T) at baseline (T1), and 150 adults repeated the LDEQ-T after an 8-week delay (T2). Test-retest reliability for self-reported dance training frequency, duration, and PA intensity was analyzed for the LDEQ-T between T1 and T2. Reliability for the LDEQ-T item on total years of professional dance training was assessed using intraclass correlation coefficients (ICCs; 2-way, random effects); linearly weighted kappa (𝜿_w_) was used for ordinal variables on dance class duration, intensity, and frequency; an unweighted kappa (𝜿) statistic was used to test the reliability of 3 dance training exposure groups (no or low, homogenous, or variable lifetime exposure to professional dance training). The significance level was set a priori with α=.05.

**Results:**

Adults reported having no or low (n=92), homogenous (n=196), or variable (n=85) lifetime exposures to professional dance classes at T1. Among adults who self-reported homogenous exposures to dance training throughout the lifespan, years of dance training were found to be reliable across all age periods (ICC range 0.83-0.94); as were weeks/month, months/year, and hours/week of dance training (𝜿_w_ range 0.35-0.61); but not any measure of PA intensity. Among adults who self-reported variable exposures to dance training, years of dance training across all age periods (ICC=0.70-0.96); classes/week, weeks/month, months/year, and hours/week (𝜿_w_ range 0.32-0.56); and some estimates of PA intensity (𝜿_w_ range 0.30-0.41) were found to be sufficiently reliable.

**Conclusions:**

The LDEQ-T appears to be a reliable instrument for quantifying lifetime participation in professional dance training among adults with self-reported low, homogenous, or variable exposures to professional dance classes throughout the lifespan. Among adults, researchers may surveil lifetime exposure to dance training in early childhood, childhood, adolescence, young adulthood, and middle or older adulthood using the LDEQ-T.

## Introduction

Dancing is positively associated with physiological, psychological, cognitive, and functional health outcomes across the lifespan [1-6]. Participation in dance refers to any of a wide range of unique or codified physical activity (PA) behaviors (eg, walking, running, sitting, standing, and jumping) performed with artistic or aesthetic intent [7]. It is widely known that PA dose—the product of the frequency, duration, and intensity of an exposure to a given mode of PA—is associated with health outcomes [8]. To that end, engaging in dance behavior, when compared to specific non-dance PA behaviors, has been reported to confer especial health benefits such as lower risk for cardiovascular disease mortality [9] and improve flexibility and balance [10]. However, additional research is needed to further extend available measures that are specifically designed to surveil lifetime participation in dance behavior from a lifespan development perspective.

Some existing long-term PA recall surveys on lifetime PA participation include items on dance and provide general estimates of the total number of years spent engaged in dance [11,12], and a subset of these instruments provides self-reported estimates on the PA intensity of dance exposures throughout the lifespan [11]. Each of these instruments can be used to obtain global estimates of the total number of years spent participating in dance across solo, professional, and social contexts. However, studies show that the PA intensity of dance may depend, in part, on the conditions under which dancing is performed or upon participant-level factors. For example, a study on the PA intensity of modern dance across class, rehearsal, and performance contexts reported dance intensities that ranged from 2.9 metabolic equivalents (METs) to 7.1 METs depending upon the context in which the dance behavior was performed [13] Another study conducted in young to older adults reported the mean intensity of solo, free-form dancing with and without music ranged from 5.6 METs to 7.5 METs and that PA intensity during dance was inversely associated with age and BMI [14]. Given that the PA intensity of dance may vary when performed across different contexts, in addition to its inverse relationship with age, the need for self-report instruments that are expressly designed for quantifying context-specific exposures to dance across the lifespan is clear. Relatedly, studies show that lifetime participation in professionally led dance classes may be interrupted for variable periods of time across the lifespan due to injury [15], for example, or due to other barriers such as insufficient resources available to pay for dance classes [16]. Moreover, some research also indicates that early developmental exposures to professionally led dance classes may be negatively associated with psychological health outcomes during adulthood in some individuals [17]. Together, these studies point toward the need for context-specific measures of lifetime dance participation that allow researchers to specifically account for exposures to professionally led dance classes, while also accounting for potential variability in professional dance training exposures across the lifespan due to factors such as barriers to access.

Though some available measures of lifetime participation in PA provide the means for estimating years of dance participation generally (ie, undifferentiated exposures to dance across solo, professional, and social contexts), studies that aim to specifically quantify exposures to professionally led dance classes at different periods across the lifespan require the development of additional measures. Dance behavior, as a term, is inclusive of dance across any context (eg, solo dancing, dancing in rehearsal and performance, or dancing socially). Professionally led dance classes refer specifically to exposures to dance that are facilitated by a professional. With specific data on exposures to dance in professionally led class environments across the lifespan, researchers can better understand how the context in which dance behavior occurs relates to markers of health. Further, more granular data on professionally led dance classes with regard to chronological age and the timing of dance exposures across the lifespan may extend future analyses on the benefits of dance for health from a developmental perspective [18]. The design of a flexible instrument for quantifying exposures to professionally led dance classes across developmental periods would thus facilitate future research on dance and health from a lifespan development perspective and augment measures of lifetime participation in PA. Therefore, the purpose of this study was to develop and determine the reliability of a new PA questionnaire, the Lifetime Dance Exposure Questionnaire for Professional Training (LDEQ-T), specifically designed to surveil exposures to professionally led dance classes throughout the lifespan among adults.

## Methods

### Participants

Adults aged 18 years and older were recruited to participate in this research study. Prospective participants were recruited via flyers, social media (ie, Facebook [Meta] and Twitter), email, and word of mouth. Flyers were posted in the Pioneer Valley of Massachusetts and via social media. Within the Pioneer Valley, flyers were posted in dance studios, senior centers, at local community events, and within local businesses that were not directly associated with dance (eg, coffee shops or martial arts training venues). The link to the questionnaire was included in emails that the lab sent as part of a quarterly newsletter. Research team members shared information about the study informally throughout their personal networks and in social settings (ie, word of mouth).

### Procedures

Participants were asked to complete a brief sociodemographic questionnaire online via REDCap (Research Electronic Data Capture; Vanderbilt University) [19]. Participants were then asked to complete the novel LDEQ-T. After 8 weeks, participants received an email invitation via REDCap to complete the LDEQ-T for a second time. Participants received reminder emails to complete the LDEQ-T for 6 weeks or until they completed the questionnaire. Prior studies that have evaluated the reliability of self-report questionnaires for measuring lifetime exposures to PA have delayed readministering the instrument for intervals of 1‐8 weeks [12,20,21], with some studies extending the delay period for up to 8 months [11].

### Sample Size Considerations

In considering the final study sample size, it was determined that at least thirty adults [22] should complete the LDEQ-T at the second time point.

### Materials

#### Sociodemographic Questionnaire

Self-reported age in years and biological sex were collected using a brief online questionnaire.

#### LDEQ-T

##### Development

The LDEQ-T is an original questionnaire designed by an expert (AKM) in dance, dance education, and movement science to specifically quantify self-reported participation in professionally led dance classes throughout the lifespan.

##### Conceptual Framework

As stated previously, PA dose is defined as the product of the intensity, duration, and frequency of a given PA exposure [8]. It follows that the LDEQ-T includes items on the PA intensity, duration, and frequency of professionally led dance classes taken throughout life.

##### Item Generation and Draft Questionnaire

The questionnaire assesses total years of dance training, the duration, frequency, and intensity of dance exposures, and it categorizes respondents into 1 of 3 dance exposure groups based upon total dance training volume and regularity. The original questionnaire had a total of 24 items; however, the number of items differs for respondents based upon branching logic [23]. Respondents categorized as having low exposure complete a single-item questionnaire; respondents categorized as having homogenous exposure complete a 13-item questionnaire; respondents categorized as having variable exposure complete a 24-item questionnaire. Each exposure group is described further below.

###### Exposure Groups

Among those who report that they have completed ≥40 professionally led dance classes, the LDEQ-T accounts for potential variability in dance participation throughout the lifespan by asking respondents to identify whether there were any major discontinuities during their participation in professionally led dance classes throughout life. Individuals who self-report that they have completed <40 professionally led dance classes conclude the questionnaire once they have completed the first questionnaire item.

####### Low Exposure

A single item on the questionnaire (ie, “Have you participated in 40 or more professionally led dance classes in your life? For reference, participating in 40 dance classes is approximately equal to taking a dance class once a week, every week, for about 10 months.”) was used to identify individuals with low exposure to professional dance training throughout life. The response format is a radio button [23] with options of “YES” and “NO” only. Respondents who self-report that they have completed <40 professionally led dance classes throughout life are categorized as individuals with low exposure to professionally led dance classes. The <40 class cut point was selected as a meaningful threshold that would represent having completed <1 year of dance training (ie, less than one class per week in a given year, assuming that annual curricula in a dance studio for children and adolescents may typically extend across a training period of ~10 months).

####### Homogenous Exposure

A single item on the questionnaire (ie, “Thinking only of the years you participated in dance classes on a regular basis, describe the usual amount of time that you spent taking dance classes year-to-year. Taking dance classes on a regular basis means participating in dance classes for 90 minutes or more per week, each week. While taking dance classes on a regular basis, the amount of time I spent taking dance classes year-to-year was.”) was used to identify individuals whose exposure to professional dance training throughout life was consistent within and across dance training periods. The response format was a radio button with options of “about the SAME each year” and “much MORE on some years and much LESS on other years” only. Respondents who self-report engaging in dance classes consistently throughout life are categorized as individuals with homogenous exposure to professionally led dance classes.

####### Variable Exposure

Individuals who self-report discontinuities in dance class engagement throughout life (ie, individuals who reported taking dance classes “much MORE on some years and much LESS on other years”) are categorized as individuals with variable exposures to professionally led dance classes.

### Total Years

The LDEQ-T asks respondents to identify each year in which they participated in dance classes that were led by a teacher from early childhood (age <2 years) to older adulthood (age >85 years). Using checkboxes [23], respondents are asked to select the checkbox next to each year that they engaged in professionally led dance classes. Individuals in the “Homogenous Exposure” group complete the total years item one time; those in the “Variable Exposure” group complete the total years item twice—once for the years in which they engaged in “MORE” dance classes than other years and once for the years in which they engaged “LESS” in taking dance classes. Questions on total years of participation were based upon the Lifetime Physical Activity Questionnaire [11], which asks respondents to select the year they began and stopped participating in each given mode of PA.

### Duration, Frequency, and Intensity

Finally, the questionnaire asks respondents to self-report the duration and PA intensity of the professionally led classes they attended. The format for these questions used drop-down menus and absolute open-ended quantifiers [23]. Individuals in the “Homogenous Exposure” group complete each item on the duration, intensity, and frequency one time; those in the “Variable Exposure” group complete each item on duration, intensity, and frequency twice—once for the years in which they engaged in “MORE” dance classes than other years and once for the years in which they engaged “LESS” in taking dance classes. Questions on duration, PA intensity, and frequency were based upon the Modifiable Activity Questionnaire and Godin-Shephard Leisure-Time PA questionnaire [24-26]. Questions on the duration and frequency of taking professionally led dance classes were fashioned after the Modifiable Activity Questionnaire, which asks respondents to report the number of times per month and the average number of minutes spent each time one engages in a given mode of PA. Questions on the perceived PA intensity of professionally led dance classes were fashioned after the Godin-Shephard Leisure-Time PA questionnaire, which asks respondents to report the total number of 15-minute bouts per week that they engaged in exercise at self-reported strenuous, moderate, and mild or light levels of effort. Examples of PA modes provided in the LDEQ-T for each level of effort were kept largely consistent with those included in the original Godin-Shepard Leisure-Time PA questionnaire, with the inclusion of dance-related behaviors. LDEQ-T items on duration, intensity, and frequency appear below:

- Duration: hours per week spent taking dance classes (drop-down menu).
- Frequency: class sessions per week (absolute open-ended quantifier; min: 1; max: 40), weeks per month (drop-down menu), and months per year (drop-down menu).
- Intensity: (1) Minutes per class spent dancing at a strenuous effort level (absolute open-ended quantifier; min: 1; max: 240); (2) Minutes per class spent dancing at a moderate effort level (absolute open-ended quantifier; min: 1; max: 240); (3) Minutes per class spent dancing at a mild or light effort level (absolute open-ended quantifier; min: 1; max: 240); (4) Times per week spent dancing in class for about 15 minutes at a strenuous effort level (absolute open-ended quantifier; min: 1; max: 160); (5) Times per week spent dancing in class for about 15 minutes at a moderate effort level (absolute open-ended quantifier; min: 1; max: 160); and (6) Times per week spent dancing in class for about 15 minutes at a mild or light effort level (absolute open-ended quantifier; min: 1; max: 160).

### Statistical Analyses

Data were analyzed in MATLAB R2024a (The MathWorks, Inc). Descriptive statistics are reported as mean (SD), median (IQR), and frequencies (percentages). LDEQ-T test-retest reliability was evaluated using data collected at time point 1 (week 0) and time point 2 (week 8+). To do this, the item-level reliability of the LDEQ-T was evaluated across time points using intraclass correlation coefficients (ICCs) and Cohen kappa coefficients. ICCs (2-way, random effects) were used to evaluate whether LDEQ-T–derived estimates were sufficiently reliable (ICC≥0.70) for total years of dance training and years of training by age group (ie, early childhood, childhood, adolescence, young adulthood, and middle-age to older adulthood). Categories of low, moderate, and high exposure were respectively developed for the self-reported frequency (classes/wk, wk/mo, and mo/y) of taking professionally led dance classes and the duration (h/wk) of classes using tertiles. Similarly, tertiles were used to bin self-reported minutes spent engaged in each PA intensity while in class into categories of low, moderate, and high exposure. Finally, tertiles were used to bin the self-reported number of 15-minute bouts spent dancing in each PA intensity into categories of low, moderate, and high exposure. Reliability for each respective categorical measure was then determined using linearly weighted kappa coefficients (𝜿_w_) to account for the ordinal scale of the data, and unweighted kappa coefficients (𝜿) were reported otherwise [27,28].

To produce a final version of the LDEQ-T, any item that was found to have insufficient reliability was removed from the instrument following the item-level reliability analyses. The final version of the LDEQ-T can be scored simply by calculating the sum of the total years spent taking dance classes over any age period, and by applying the tertile-based cut points to any items on class duration, frequency, and intensity to categorize respondents into low, moderate, and high exposure groups for each respective item. The questionnaire may also be scored with the LDEQ-T companion app [29], which was developed by the researcher (AKM) using MATLAB and is available to be downloaded. The significance level in all models was set a priori with α=.05.

### Ethical Considerations

The study was approved by the Institutional Review Board at the University of Massachusetts Amherst (IRB #2495), and all participants provided written informed consent. Data were collected between February 2022 and August 2023, data were deidentified, and the study was conducted entirely online.

## Results

### Overview

**Figure 1.**
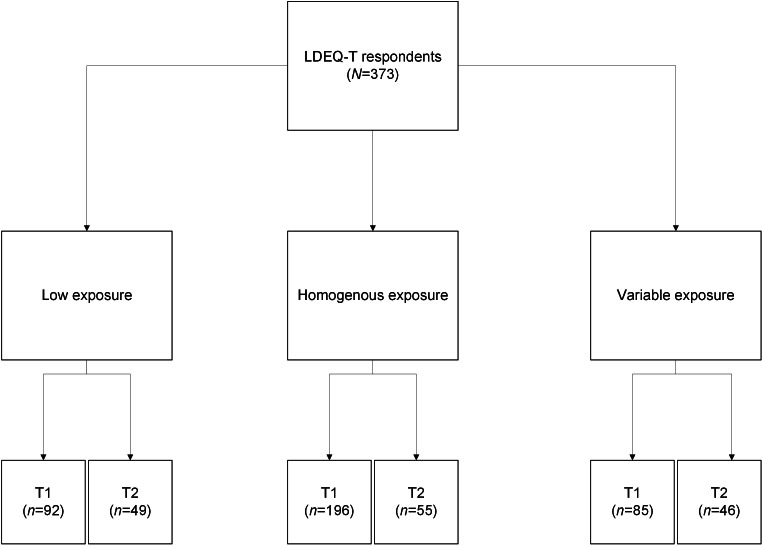
Responses to the Lifetime Dance Exposure Questionnaire for Professional Training note: Figure 1 shows the total number of Lifetime Dance Exposure Questionnaire for Professional Training responses collected at baseline (timepoint 1; T1) and after an 8+ week delay (timepoint 2; T2) among adults who self-reported having low exposure to professionally led dance classes throughout life, in addition to those who reported homogenous or variable exposures to dance training year-to-year. LDEQ-T: Lifetime Dance Exposure Questionnaire for Professional Training.

**Figure 2.**
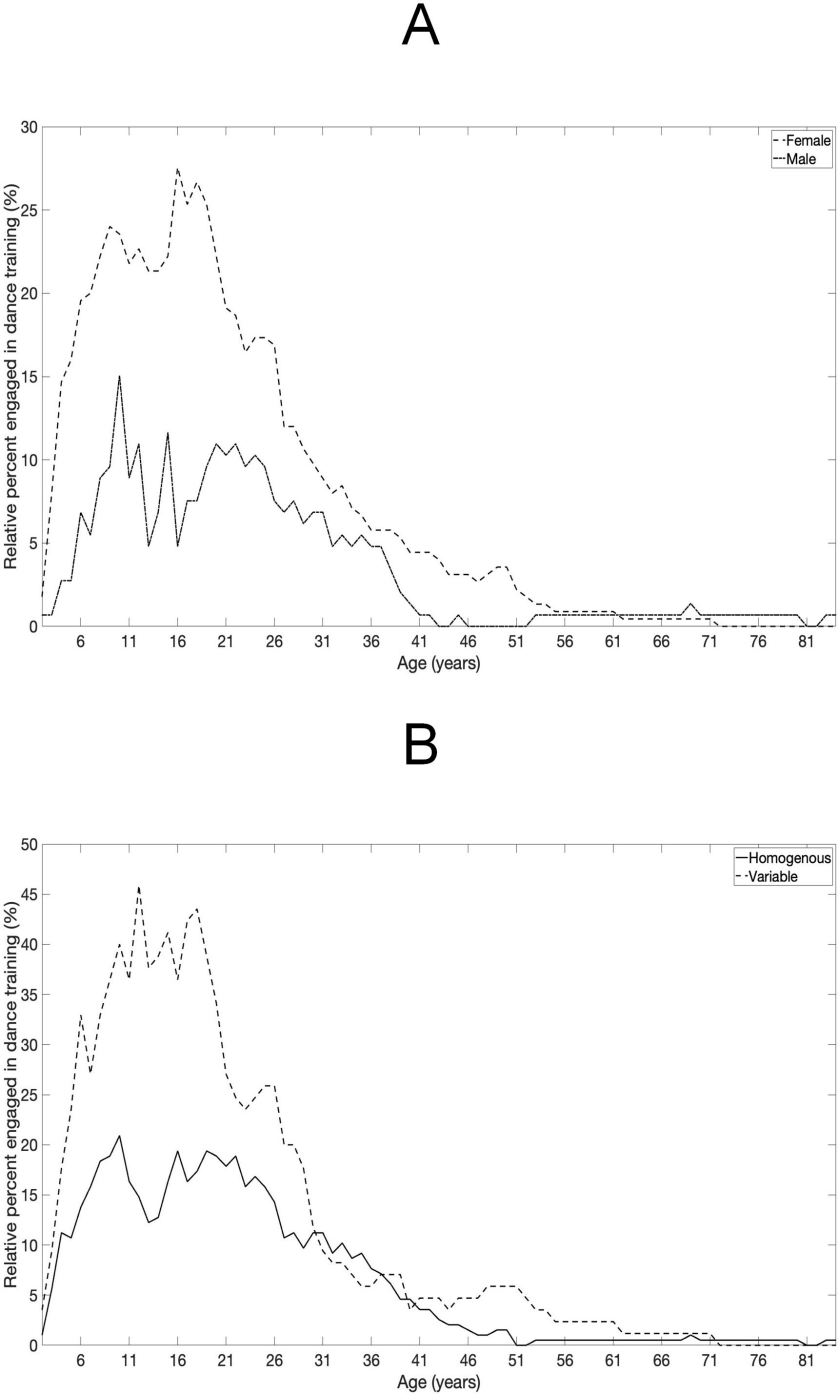
Participation in professional dance classes across the lifespan by sex (A) and by dance exposure pattern (B) among adults aged 18 to 84 years as reported using the Lifetime Dance Exposure Questionnaire (LDEQ-T). Note: Figure 2A shows the relative percentage of adults who self-reported participating in professionally led dance classes across the lifespan for female and male LDEQ-T respondents. Figure 2B shows the relative percentage of adults who self-reported participating in professionally led dance classes across the lifespan by exposure pattern (ie, homogenous or variable exposures to dance).

A total of 373 adults (Figure 1) completed the LDEQ-T at timepoint one (T1). Of the overall sample, 365 adults reported their age (range 18‐84 y) at T1; 81.9% (n=299) were young adults (aged 18‐39 years; median 27, IQR 23-30) and 18.1% (n=66) were middle age to older adults (aged 40‐84 y; median 49, IQR 43-57). A total of 150 adults repeated the questionnaire at time point 2 (T2). LDEQ-T test-retest reliability between T1 and T2 was sufficient for the self-reported lifetime exposure categories of low exposure, homogenous exposure, and variable exposure [𝜿(SE)=0.53 (0.06), 95% CI 0.42-0.64; *P*<.001]. Figure 2A shows self-reported dance training across the lifespan, by biological sex, using LDEQ-T responses collected at T1. The range of self-reported years of participation in professionally led dance classes ranged from 0 to 68 total years at T1.

### Low Exposure to Dance Training

A total of 92 adults (mean age 32, SD 12.8 y; biological sex: female 52.2%, 48/92, male 47.8%, 44/92) self-reported engaging in <40 professionally led dance classes throughout life, and of these, a total of 49 adults completed the LDEQ-T at T2. The percent agreement between T1 and T2 for those who self-reported low exposure to dance training was 73.5%—among adults who reported low exposure to dance training at T1, the range of self-reported total years of dance training at T2 was 0 to 16 years.

### Homogenous Exposure to Dance Training

A total of 196 adults (mean age 30, SD 10.7 y; biological sex: female 60.7%, 119/196, male 38.8%, 76/196, not reported 0.5%, 1/196) indicated that they experienced homogenous exposures to professionally led dance classes throughout life when completing the LDEQ-T at T1, and of these n=55 adults completed the LDEQ-T at T2. Figure 2B shows self-reported exposures to professionally led dance classes across the lifespan for adults who reported homogenous exposures to dance training at T1.

Self-reported years of dance training were found to be sufficiently reliable (ICC range 0.83-0.94) for those who self-reported experiencing homogenous exposures to professionally led dance classes across the lifespan (Table 1). As Table 2 shows, the frequency and duration of self-reported professional dance classes were also found to be reliable when assessed in terms of weeks/month, months/year, and hours/week (*𝜿*_w_ range 0.35-0.61). Remaining items on frequency, duration, and intensity had low kappa coefficients and were excluded from the final version of the LDEQ-T due to insufficient reliability (*P*>.05). These items included average class sessions per week (𝜿_w_=0.23); average minutes of dance at strenuous (𝜿_w_=0.18), moderate (𝜿_w_=0.19), and mild or light levels of perceived exertion (𝜿_w_=0.17) per class; and average times per week spent dancing for about 15 minutes in class at strenuous (𝜿_w_=0.21), moderate (𝜿_w_=0.10), and mild or light (𝜿_w_=0.05) levels of perceived exertion.

**Table 1.** Lifetime Dance Exposure Questionnaire for Professional Training reliability (homogenous exposure): years of training by age group. Note: Table 1 shows the mean (SD), and intraclass correlation coefficients for years of dance training for five age groups across timepoint 1 (T1) and timepoint 2 (T2) among adults who reported having homogenous exposures to professionally led dance classes throughout life.

Years of training	T1, mean (SD)	T2, mean (SD)	ICC^a^ (95% CI)
Total lifetime training (n=55)	7.3 (8.9)	6.7 (8.5)	0.89 (0.85-0.93)
Early childhood training (age >2 to 5 years)	0.5 (1)	0.5 (1.1)	0.81 (0.75-0.86)
Childhood training (age 6 to 11 years)	1.6 (2.1)	1.7 (2.2)	0.91 (0.89-0.93)
Training during adolescence (age 12 to 17 years)	1.6 (2.1)	1.7 (2.3)	0.89 (0.85-0.93)
Training during young adulthood (age 18 to 39 years)	2.5 (4.8)	2.4 (4.6)	0.94 (0.92-0.95)
Training during middle and older adulthood (age >40 years; n=28)	0.8 (2.5)	0.5 (1.8)	0.83 (0.73-0.93)

a ICC: intraclass correlation coefficients.

**Table 2.** Lifetime Dance Exposure Questionnaire for Professional Training reliability (homogenous dance exposures): Dance class frequency and duration tertiles. Note: Table 2 summarizes the percentage of participants within low, moderate, and high exposure tertiles for LDEQ-T items on dance exposure frequency (weeks/months; months/year) and duration (hours/week) at baseline (T1) and after an 8+ week delay (T2). Italicized numbers show corresponding tertiles for each respective item on frequency and duration.

Measures	Low, n (%)	Moderate, n (%)	High, n (%)	𝜿_w_	SE (95% CI)	*P* value
Weeks per month (n=55)	*1-2*	*3*	*4*	0.61	0.11 (0.40-0.82)	<.001
T1	18 (32.7)	5 (9.1)	32 (58.2)			
T2	24 (43.6)	4 (7.3)	27 (49.1)			
Months per year (n=55)	*1-5*	*6-11*	*12*	0.35	0.14 (0.09-0.62)	.009
T1	14 (25.4)	21 (38.2)	20 (36.4)			
T2	26 (47.3)	19 (34.5)	10 (18.2)			
Hours per week (n=55)	*1-3*	*4-10*	*>10*	0.38	0.15 (0.09-0.67)	.01
T1	22 (40)	22 (40)	11 (20)			
T2	35 (64.8)	13 (24.1)	6 (11.1)			

### Variable Exposure to Dance Training

A total of 85 participants completed the LDEQ-T at T1 and indicated having variable exposure to dance training throughout life (mean age 30, SD 13.4 y; biological sex: female 68.2%, 58/85, male 30.6%, 26/85, not reported 1.2%, 1/85), and of these, a total of 46 adults completed the LDEQ-T at T2. Figure 2B shows self-reported dance training across the lifespan for adults who reported variable exposure to dance training at T1. Self-reported years of dance training were found to be sufficiently reliable (ICC range 0.70-0.96) for those who reported experiencing variable exposures to dance training across the lifespan (Table 3).

**Table 3.** Lifetime Dance Exposure Questionnaire for Professional Training reliability (variable dance exposure): Years of training by age group. Note: Table 3 shows the mean (SD), and intraclass correlation coefficients (ICC) for years of dance training for five age groups across timepoint 1 (T1) and timepoint 2 (T2) among adults who reported having variable exposure to professionally led dance classes throughout life.

Years of training	T1, mean (SD)	T2, mean (SD)	ICC^a^ (95% CI)
Total lifetime training (n=46)	13.6 (13.1)	11.6 (10.4)	0.93 (0.89-0.96)
Early childhood training (age >2 to 5 years)	0.8 (1.2)	0.7 (1.2)	0.80 (0.76-0.84)
Childhood training (age 6 to 11 years)	2.8 (2.6)	2.6 (2.6)	0.90 (0.88-0.92)
Training during adolescence (age 12 to 17 years)	3 (2.6)	2.8 (2.6)	0.93 (0.90-0.95)
Training during young adulthood (age 18 to 39 years)	5.6 (6.2)	5 (5.7)	0.96 (0.94-0.97)
Training during middle and older adulthood (age >40 years; n=18)	1.2 (4.4)	0.5 (1.8)	0.70 (0.56-0.84)

a ICC: intraclass correlation coefficients.

#### High Exposure Years

As shown in Table 4, the frequency and duration of self-reported professional dance classes were also found to be reliable when assessed in terms of classes/week, weeks/month, months/year, and hours/week (𝜿_w_ range 0.38-0.56).

**Table 4.** Lifetime Dance Exposure Questionnaire for Professional Training reliability (variable dance exposure): Dance class frequency and duration tertiles during high exposure years. Note: Table 4 summarizes the percentage of participants within low, moderate, and high exposure tertiles for items on dance exposure frequency (weeks/months; months/year) and duration (hours/week) during their high dance exposure years at baseline (T1) and after an 8+ week delay (T2). Italic values represent corresponding tertiles for each respective item on frequency and duration.

Measures	Low	Moderate	High	𝜿_w_	SE (95% CI)	*P* value
Classes per week (N=46)	*1-2*	*3-5*	*>5*	0.56	0.13 (0.31-0.81)	<.001
T1	7 (15.2)	15 (32.6)	24 (52.2)			
T2	19 (41.3)	8 (17.4)	19 (41.3)			
Weeks per month (N=46)	*1-2*	*3*	*4*	0.38	0.16 (0.07-0.69)	.016
T1	5 (10.9)	6 (13)	35 (76.1)			
T2	15 (32.6)	3 (6.5)	28 (60.9)			
Months per year (N=46)	*1-8*	*9-11*	*12*	0.47	0.14 (0.20-0.74)	<.001
T1	8 (17.4)	20 (43.5)	18 (39.1)			
T2	20 (43.5)	14 (30.4)	12 (26.1)			
Hours per week (N=46)	*1-5*	*6-13*	*>13*	0.55	0.13 (0.30-0.81)	<.001
T1	16 (34.8)	15 (32.6)	15 (32.6)			
T2	22 (47.8)	10 (21.8)	14 (30.4)			

All but 2 items pertaining to dance intensity were reliable, summarized in Table 5 (𝜿_w_: 0.33-0.42). Items with low kappa coefficients and insufficient reliability (*P*>.05) were average class minutes spent dancing at mild or light levels of perceived exertion (𝜿_w_=0.17) and average times per week spent dancing for about 15 minutes in class at strenuous levels of perceived exertion (𝜿_w_=0.23), which were excluded from the finalized LDEQ-T.

**Table 5.** Lifetime Dance Exposure Questionnaire for Professional Training reliability (variable dance exposure): Time spent engaged in dance class at mild to strenuous physical activity intensities—tertiles during high exposure years. Note: Table 5 summarizes the percentage of participants within low, moderate, and high tertiles for items on dance intensity (minutes and number of 15-minute bouts of dancing per week at different levels of perceived exertion) during their high dance exposure years at baseline (T1) and after an 8+ week delay (T2). Italic values represent corresponding tertiles for each respective item on intensity.

Measures	Low	Moderate	High	*κ*_w_	SE (95% CI)	*P* value
Strenuous minutes (n=46)	*1‐19*	*20‐33*	*>33*	0.33	0.15 (0.04-0.62)	.027
T1	11 (23.9)	16 (34.8)	19 (41.3)			
T2	21 (46.7)	14 (31.1)	10 (22.2)			
Moderate minutes (n=46)	*1‐19*	*20‐33*	*>33*	0.41	0.14 (0.13-0.68)	.004
T1	12 (26.1)	14 (30.4)	20 (43.5)			
T2	21 (45.7)	12 (26.1)	13 (28.2)			
Moderate bouts (n=46)	*1‐2*	*3‐6*	*>6*	0.36	0.15 (0.06-0.65)	.019
T1	13 (28.3)	17 (36.9)	16 (34.8)			
T2	19 (41.3)	17 (37)	10 (21.7)			
Mild or light bouts (n=46)	*1‐2*	*3‐6*	*>6*	0.42	0.15 (0.13-0.71)	.005
T1	14 (30.43)	18 (39.13)	14 (30.44)			
T2	19 (41.3)	17 (37)	10 (21.7)			

#### Low Exposure Years

As shown in Table 6, the frequency and duration of self-reported professional dance classes were also found to be reliable when assessed in terms of classes/week, weeks/month, months/year, and hours/week (𝜿_w_ range 0.32-0.43) during self-reported less active dance training years.

**Table 6.** Lifetime Dance Exposure Questionnaire for Professional Training reliability (variable dance exposure): Dance class frequency and duration tertiles during low exposure years. Note: Table 6 summarizes the percentage of participants within low, moderate, and high exposure tertiles for items on dance exposure frequency (weeks/months; months/year) and duration (hours/week) during their low dance exposure years at baseline (T1) and after an 8+ week delay (T2). Italic values represent corresponding tertiles for each respective item on frequency and duration.

Measures	Low	Moderate	High	𝜿_w_	SE (95% CI)	*P* value
Classes per week (N=46)	*1*	*2*	*3*	0.33	0.15 (0.06-0.62)	.021
T1	15 (32.6)	13 (28.3)	18 (31.1)			
T2	19 (45.7)	10 (21.7)	15 (32.6)			
Weeks per month (N=46)	*1*	*2-3*	*>3*	0.33	0.15 (0.03-0.63)	.033
T1	5 (10.8)	13 (28.3)	28 (60.9)			
T2	15 (32.6)	10 (21.7)	21 (45.7)			
Months per year (N=46)	*1-5*	*6-10*	*>10*	0.43	0.14 (0.16-0.70)	.002
T1	9 (19.6)	7 (15.2)	30 (65.2)			
T2	19 (41.3)	4 (8.7)	23 (50)			
Hours per week (N=46)	*1-2*	*3-6*	*>6*	0.32	0.16 (0.01-0.62)	.043
T1	17 (37)	15 (32.6)	14 (30.4)			
T2	25 (54.3)	16 (34.8)	5 (10.9)			

Two items on perceived dance intensity were sufficiently reliable for less active dance training years, as summarized in Table 7 (𝜿_w_ range 0.30-0.31). Remaining items with low kappa coefficients and insufficient reliability (*P*>.05) were excluded from the final LDEQ-T, which were average class minutes spent dancing at strenuous levels of perceived exertion (𝜿_w_=0.30) and average times per week spent dancing for about 15 minutes in class at strenuous (𝜿_w_=0.23), moderate (𝜿_w_=0.24), and mild or light (𝜿_w_=0.21) levels of perceived exertion.

**Table 7.** Lifetime Dance Exposure Questionnaire for Professional Training reliability (variable dance exposure): Minutes spent engaged in dance class at mild or light and moderate physical activity intensities—tertiles during low exposure years. Note: Table 7 summarizes the percentage of participants within low, moderate, and high tertiles for items on dance intensity (minutes of dancing per week at different levels of perceived exertion) during their low dance exposure years at baseline (T1) and after an 8+ week delay (T2). Italic values represent corresponding tertiles for each respective item on intensity.

Measures	Low	Moderate	High	𝜿_w_	SE (95% CI)	*P* value
Moderate minutes (N=46)	*1‐14*	*15‐33*	*>33*	0.30	0.15 (0.001-0.598)	.049
T1	9 (19.6)	20 (43.5)	17 (36.9)			
T2	21 (45.7)	15 (32.6)	10 (21.7)			
Mild or light minutes (N=46)	*1‐14*	*15‐23*	*>23*	0.31	0.14 (0.03-0.59)	.031
T1	12 (26.1)	15 (32.6)	19 (41.3)			
T2	25 (54.4)	10 (21.7)	11 (23.9)			

### LDEQ-T Refinement and Companion App

#### Questionnaire Refinement

Items with insufficient reliability were removed from the original 24-item version of LDEQ-T. The final LDEQ-T (18 items) can be downloaded as a *.pdf and as a REDCap “Data Dictionary” [29]. The total number of items for each exposure group in the final version of the questionnaire was 1, 6, and 18 for those in the low, homogenous, and variable exposure groups, respectively. Field notes show that the questionnaire may be completed in 1‐15 minutes.

#### LDEQ-T Companion App

The LDEQ-T app (KinetiWave) allows users to select a spreadsheet with LDEQ-T responses, as exported from REDCap, and it returns a summary of the total number of years spent engaged in professional dance training, as well as a summary of the years spent engaged in dance classes across each age group from early childhood to middle and older adulthood. In addition, the LDEQ-T companion app applies the empirically derived, tertile-based cutoff points to all LDEQ-T items on the self-reported duration, frequency, and perceived intensity of professionally led dance classes. An example summary report can be downloaded with the LDEQ-T app [29].

## Discussion

### Overview

This study aimed to determine the reliability of a new questionnaire, the LDEQ-T, which was specifically designed to surveil lifetime exposures to professionally led dance classes among adults. Analyses of the LDEQ-T revealed that it is a reliable questionnaire for assessing lifetime participation in professionally led dance classes among adults aged 18 to 84 years. When the LDEQ-T was used to assess dance training among adults who self-reported experiencing homogenous exposures to professionally led dance classes throughout life, self-reports on the frequency and duration of dance classes, but not the PA intensity of classes, were found to be reliable. Among adults who self-reported experiencing variable exposure to professionally led dance classes across life, self-reports on the frequency, duration, and the PA intensity of dance classes were respectively found to be reliable for LDEQ-T items depending upon the overall level of self-reported dance training activity each year. Overall, results show the LDEQ-T is a reliable instrument for assessing lifetime participation in professionally led dance classes among adults with a range of prior exposures to dance training.

### Principal Results

In a sample of adults aged 18 to 84 years, the LDEQ-T provided reliable estimates of the total number of years in which respondents engaged in professionally led dance classes across the lifespan. Among a cohort of adults with prior exposures to dance training, LDEQ-T reliability estimates were observed to be good to very good (ICC>0.89) for self-reported total years of experience in professionally led dance classes. Reliability estimates for the LDEQ-T were also observed to be sufficient when years of dance training were analyzed by dance exposure by age groups, early childhood, adolescence, young adulthood, middle or older adulthood (ICC>0.70). Study results showed that the LDEQ-T can be used as a reliable measure of the total number of years in which adults have engaged in professionally led dance classes, in addition to providing a measure of age-specific exposures to dance training across the lifespan. Prior studies that have evaluated the reliability of lifetime PA questionnaires report ICCs in the range 0.67‐0.89 [11,12]. Thus, the reliability of the LDEQ-T is comparatively strong, and future studies may use the LDEQ-T to obtain self-reports of the total number of years one has engaged in professionally led dance classes across the lifespan, as well as assess exposure to professionally led dance classes by developmental age groups.

Reliability for LDEQ-T items about the PA intensity of dance classes was found to be sufficient among adults who self-reported experiencing variable exposure to dance training throughout life. Specifically, during years that adults reported as being more active dance training years, reliability was found to be sufficiently high for items on self-reported time spent at strenuous and moderate (𝜿_w_: 0.33-0.41) levels of perceived effort and on self-reported weekly bouts of activity performed at moderate and mild or light (𝜿_w_: 0.36-0.42) levels of perceived effort. Similarly, for the years adults reported they were less engaged in dance training, reliability was found to be sufficient [30] for items on self-reported minutes spent at moderate and mild or light levels of perceived effort (𝜿_w_: 0.30-0.31). For adults who reported experiencing homogenous exposures to dance training throughout life, no items on the perceived intensity of dance classes were found to be sufficiently reliable. Average years of dance training experience were qualitatively higher among adults with variable exposures to dance training (13.6 y) versus those with homogenous exposures to dance training (7.3 y). It is possible that those who participated in dance over a greater number of years were more consistently able to recall the perceived PA intensity of professionally led dance classes; however, additional research is needed to understand how lifetime PA exposure patterns may potentially affect long-term PA intensity recall.

The results of this study suggest that, among adults with variable exposures to dance training throughout life, the LDEQ-T can be used to recall and assess the self-reported perceived PA intensity of dance classes during both more active and less active dance training years. Among adults with homogenous exposures to dance training, results also show the LDEQ-T can be used to recall and assess the self-reported frequency and duration of dance classes taken throughout life. The LDEQ-T questionnaire can be obtained, along with a copy of the questionnaire items, response scale characteristics, and REDCap “Data Dictionary” as part of the LDEQ-T app download [29]. The LDEQ-T app includes a calculator for LDEQ-T responses; the app applies the tertile cut points, as presented in our study results, and returns summary statistics on professionally led dance class exposure for respondents. Work from our group showed that total years of exposure to professionally led dance classes, as measured by the LDEQ-T, were related to movement qualities associated with free-form dance in a study that used wearable devices to quantify motor behavior quality among adults aged 18 to 83 years with a range of 0 to 56 years of professional dance training [31].

### Limitations and Delimitations

A limitation of the study is that no concurrent measure of lifetime exposure to professionally led dance training was used to validate the LDEQ-T. This is because, to our knowledge, this is the first study to develop a method for surveilling exposure to professionally led dance classes throughout life. While the lack of a concurrent measure of PA may be a limitation that is typical of most studies that have developed long-term PA recall questionnaires [21,32], studies have also been previously conducted that concurrently integrated the use of wearable devices in the development of long-term PA recall instruments [33]. Importantly, a major difference between this study and prior studies that have used wearable devices to validate long-term PA recall instruments is that the LDEQ-T accounts for dance training in childhood, while prior studies that have used wearable devices to validate their questionnaire were focused only on PA in adulthood. Future studies on dance that seek to further develop surveys for long-term PA recall should consider including wearable sensors as part of the questionnaire design process to further advance PA recall-based methods [32,33]. Given that no other measure exists for expressly assessing exposures to professionally led dance classes across the lifespan, all reports on years of dance training experience that were represented in this study sample were obtained from the LDEQ-T. A delimitation of the study is that children and adolescents were not included in the sample. The study sample included young to older adults, and the item-level reliability of the LDEQ-T questionnaire was found to be sufficient across age groups. Nevertheless, it is important to note that the study sample was comprised mostly of adults aged 18 to 39 years (~82%), and adults aged 40 to 84 years comprised the remaining ~18 % of the sample. Future studies are required to determine if the LDEQ-T can be used among non-adult respondents; additional research on the LDEQ-T among older adults, especially >75 years, may be needed. Finally, the LDEQ-T does not include any open-ended questions on self-reported dance style; therefore, researchers may need to additionally allow respondents to provide qualitative feedback in order to further document dance heritage or style when characterizing lifetime dance exposure.

### Strengths

A strength of the study is that a sample of young to older adults, aged 18 to 84 years, both with and without prior dance training, were recruited to complete the LDEQ-T. The LDEQ-T provides a novel measure of dance training that allows researchers to account for variable exposures to dance training throughout the lifespan. Furthermore, the LDEQ-T offers a context-specific measure of dance training that focuses on exposure to dance in professionally led classes. In view of the promising findings presented in this study, we recommend that researchers consider using the LDEQ-T, and its companion calculator app [29], to assess exposures to professionally led dance classes across the lifespan in future studies of dance and health among adults with or without prior dance training. Finally, we present the item-level reliability for the LDEQ-T questionnaire, which enables researchers to select and use any of the LDEQ-T items that are most relevant to the purposes of their research.

### Conclusions

The LDEQ-T appears to be a reliable self-report instrument for assessing the frequency and duration of lifetime participation in professionally led dance classes among adults aged 18 to 84 years, with a range of 0 to 68 years of dance training. Adults who have experienced variable exposure to professional dance training throughout life may additionally use the LDEQ-T to reliably report the perceived intensity of dance classes. Future studies on lifetime exposures to dance training among adults may use the LDEQ-T to surveil dance exposures from a lifespan development perspective.

## Abbreviations

ICC: intraclass correlation coefficients
LDEQ-T: Lifetime Dance Exposure Questionnaire for Professional Training
MET: metabolic equivalent
PA: physical activity
REDCap: Research Electronic Data Capture
